# Primary breast lymphoma in males: Incidence, demographics, prognostic factors, survival, and comparisons with females

**DOI:** 10.3389/fsurg.2022.984497

**Published:** 2022-08-25

**Authors:** Jie Zhang, Binbin Ma, Hong Ji, Rong Guo

**Affiliations:** ^1^ Department of Plastic Surgery, The Second Hospital of Hebei Medical University, Shijiazhuang, China; ^2^ Department of Neurology, Weifang Hanting People's Hospital, Weifang, China; ^3^ Department of General Surgery, The Second Hospital of Hebei Medical University, Shijiazhuang, China; ^4^ Department of Ultrasound, Weifang People's Hospital, Weifang, China

**Keywords:** primary breast lymphoma, incidence, prognosis, survival, death cause

## Abstract

**Background:**

Primary breast lymphoma (PBL) is a rare disease condition and is mainly observed in females. No male PBL cohorts were reported previously. This study aims to investigate the incidence, clinical characteristics, prognostic factors, and survival outcomes among male PBL patients and also to perform comparisons between males and females.

**Methods:**

Patients diagnosed with PBL between 2000 and 2019 from the Surveillance, Epidemiology, and End Results (SEER) database were identified. Age-adjusted incidence rates were calculated by year and age for trend analysis. Univariate and multivariate Cox hazard proportional regression analyses were performed to identify prognostic factors. Survival comparisons were conducted using the Kaplan–Meier method and the log-rank test. The propensity matching score (PSM) method was used to balance demographics.

**Results:**

The incidence rate of 122 male PBL patients diagnosed in the period of 2000 to 2019 was 0.169 (95% CI: 0.140–2.203) per million persons, which was much lower than that of 2,543 females (1.59, 95% CI: 1.53–1.65). Clinical demographics were similar between females and males, except for lymphoma subtype distribution (*P* = 0.025). A higher age [hazard ratio (HR) = 1.08, 95% CI: 1.05–1.12, *P* < 0.001] and not receiving radiotherapy (receiving vs. not receiving: HR = 0.41, 95% CI: 0.21–0.78, *P* = 0.007) were significant risk factors associated with overall survival (OS) in males. Radiotherapy (OS: *P* = 0.023) can offer benefits in OS. Using the PSM method, we also revealed that male PBL patients had significantly worse OS and cancer-specific survival rates than females.

**Conclusions:**

This study first analyzed male patients with PBL involving incidence, clinical characteristics, and survival data. Sex disparity was also observed in the survival outcome of the disease.

## Introduction

Primary breast lymphoma (PBL) is a rare disease condition and accounts for approximately 0.5% of all breast malignancies (1, 2), which demonstrates multiple similarities to other common breast carcinomas in both clinical manifestations and radiological features (3, 4). Wiseman and Liao confirmed the definition of PBL in 1972, including the following criteria: (1) sufficient tumor tissue for histological examination; (2) no previous lymphoid malignancy at regions outside the breast; (3) lymphoma primarily presented within breast regions; and (4) close associations between lymphoma and breast tissue (5). This infrequent tumor was found especially in females, and quite uniquely, there were a few case reports in males in previous literature studies (6–23). Due to the extreme rarity and unspecific manifestations, male patients with PBL are easily misdiagnosed clinically, thus delaying the optimal treatment time window and causing great pain to patients during the period of seeking medical advice. In addition, the treatment and prognosis among them remains unclear.

To our knowledge, there was still a lack of male cohorts of PBL. In the present study, we collected the largest cohort of male patients from the Surveillance, Epidemiology, and End Results (SEER) program database and first reported the clinical analyses of male PBL involving incidence, clinical demographics, prognostic factors, and survival outcomes. Meanwhile, we also performed comparisons between male and female patients to reveal the sex differences of the rare disease.

## Methods

### Study Population

The public cancer database of SEER collected the clinical data of approximately one-third of cancer patients in the United States (https://seer.cancer.gov/seerstat/). From this database, we identified patients from 18 states who were diagnosed with primary lymphoma of the breast between 2000 and 2019 with the International Classification of Diseases for Oncology-3 (ICD-O-3) code as lymphoma that primarily occurred in the breast. This study was carried out in accordance with the Declaration of Helsinki. Because the SEER database was publicly available, all patients enrolled in our study were recorded without any recognizable identity. The Ethics Committee of Weifang People's Hospital has waived the ethical approval.

### Incidence Analysis

Although PBL is a rare occurrence, we aim to calculate the incidence rates of male and female patients with adjustments made for age by 2000 US standard population using SEER*Stat software (version 8.4.0; https://seer.cancer.gov/seerstat/). The incidence trends can be presented as the diagnosis year or age and compared between the two sexes.

### Demographic Characteristics

The clinical demographics of PBL patients were described with the year of diagnosis (2000–2019), age of diagnosis, ethnicity (White, Black, American Indian, Alaska Native, Asian, or Pacific Islander), sex, tumor laterality, Ann Arbor stage, surgical resection, radiotherapy, and chemotherapy. The follow-up-related indicators included survival time since diagnosis, survival status at the last update (November 2021), and death causes (if dead; according to ICD-10 codes for classification).

### Statistical Analysis

All statistical analyses and comparisons were made using IBM SPSS 26.0 and R software 4.1.0 (https://www.r-project.org). For continuous variables such as age [represented by the median value and interquartile range (IQR)], the Shapiro–Wilk test was first used for examining normality, and a nonparametric test (Mann–Whitney *U* test) was then performed to compare the difference between male and female patients with PBL if it did not fit the normal distribution. In addition, the *χ*^2^ test was used to compare categorical variables (Fisher's exact test if necessary).

Univariate and multivariate Cox hazard proportional regression analyses were further performed to identify the risk factors associated with the overall survival (OS) among male PBL patients. The Kaplan–Meier method was used to plot the survival curves of OS and cancer-specific survival (CSS; lymphoma-specific), and the log-rank test was used for making survival comparisons among different subgroups. The propensity matching score (PSM) method was used to balance the potential factors between males and females. A *P*-value <0.05 was considered statistically significant in this study.

## Results

### Incidence Statistics

**Figure 1:**
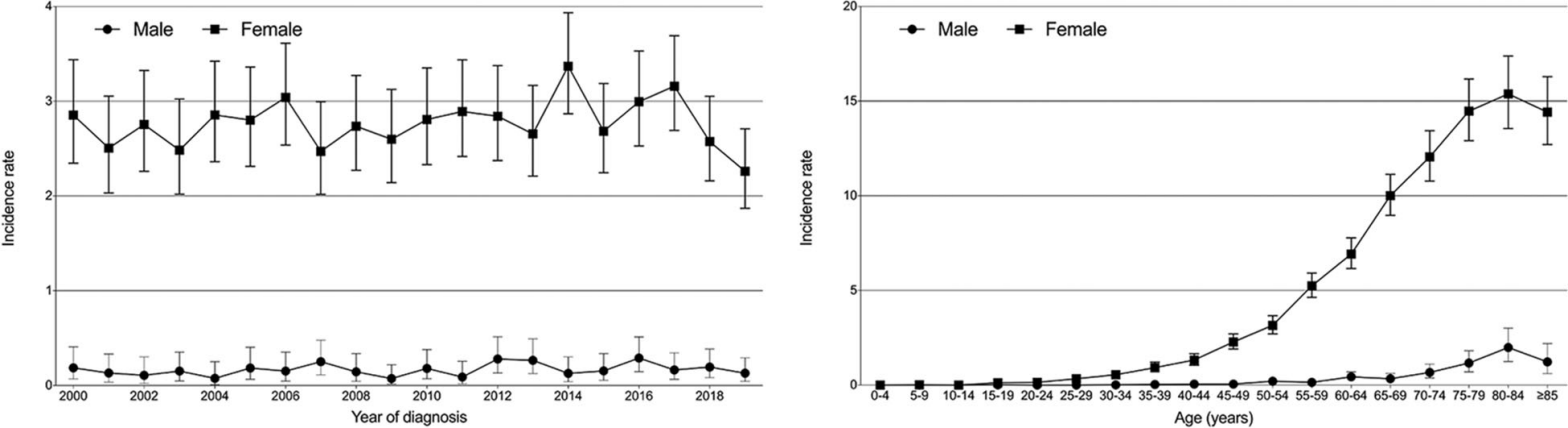
Incidence trends over the diagnosis year and age in male and female patients with primary breast lymphoma.

From the SEER database, we identified a total of 122 males and 2,543 females diagnosed with PBL between 2000 and 2019. During those 20 years, the overall age-adjusted incidence rates of PBL were 0.169 [95% confidence interval (95% CI): 0.140–2.203] per million persons for males and 1.59 (95% CI: 1.53–1.65) per million persons for females. As shown in Figure 1, none of the obvious trend changes from 2000 to 2019 was observed in both incidence rates of male and female patients. Furthermore, whether for male or female, higher incidence rates were observed in older patients, which reached a peak at the age range of 80–84 years (male: 1.99, per million, 95% CI: 1.25–3.01; female: 15.38, 95% CI: 13.55–17.39, per million).

### Clinical Characteristics

All 122 male patients with PBL included 108 (88.5%) Caucasians and 14 (11.5%) Blacks, and no other races were found in males (Table 1). The median age was 71 (IQR: 60–80) years for males, compared with 67 (57–77) years for females. Most of the male cases (*N* = 119, 97.5%) were diagnosed with non-Hodgkin’s lymphoma (NHL), which demonstrated a significantly less proportion than that of females (*N* = 2,526, 99.3%; *P* = 0.025). Actually, there were only three male patients with Hodgkin’s lymphoma (HL). Of 79 staged PBL males, 38 (48.1%) were recorded as stage I, 17 (21.5%) as stage II, 5 (6.3%) as stage III, and 19 (24.1%) as stage IV. As for treatments, 34.4% (*N* = 42) of male patients underwent surgery, 31.1% received radiotherapy, and 47.5% received chemotherapy. There was no significant difference between male and female patients with PBL among clinical characteristics, except for the lymphoma subtype.

**Table 1:** Clinical demographics among male and female patients with primary breast lymphoma.

Characteristic	Before PSM				After PSM		
	Total	Male (*N* = 122)	Female (*N* = 2543)	*P*-value	Male (*N* = 117)	Female (*N* = 232)	*P*-value
Age, years							
Median (IQR)	68 (57–77)	71 (60–80)	67 (57–77)	0.055	71 (60–80)	71 (60–81)	0.386
Race							
White	2,205	108	2,097	0.140	104	190	0.139
Black	420	14	406		13	38	
Others	40	0	40		0	4	
Year of diagnosis							
2000–2004	556	20	536	0.601	20	44	0.867
2005–2009	622	28	594		27	55	
2010–2014	728	35	693		33	56	
2015–2019	759	39	720		37	77	
Subtype							
HL	20	3	17	**0** **.** **025**	2	4	0.992
NHL	2,645	119	2,526		115	228	
Laterality							
Left	1,226	57	1,169	0.982	55	103	0.73
Right	1,311	59	1,252		53	105	
Undefined	128	6	122		6	9	
Ann Arbor stage							
Stage I	1,129	38	1,091	0.150	37	94	0.288
Stage II	325	17	308		16	22	
Stage III	94	5	89		5	5	
Stage IV	325	19	306		18	27	
Unstaged	792	43	749		41	84	
Surgery							
Not received	1,833	80	1,753	0.434	76	146	0.71
Received	832	42	790		41	86	
Radiotherapy							
Not received	1,829	84	1,745	0.957	79	157	0.977
Received	836	38	798		38	75	
Chemotherapy							
Not received	1,463	64	1,399	0.580	61	124	0.817
Received	1,202	58	1,144		56	108	

IQR, interquartile range; PSM, propensity scoring matching; HL, Hodgkin’s lymphoma; NHL, non-Hodgkin’s lymphoma.

Bold indicates *P* value < 0.05.

### Survival Outcome

**Figure 2:**
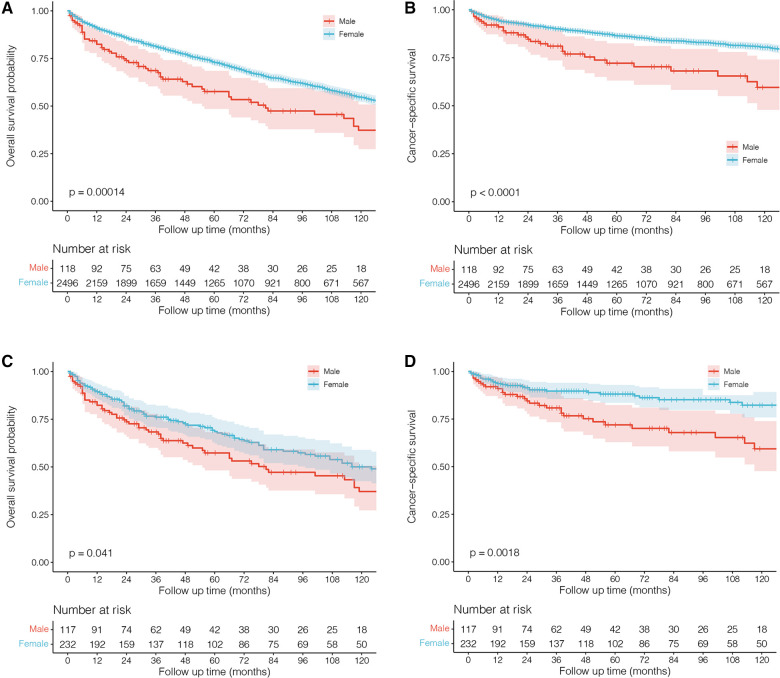
Kaplan–Meier survival curves and log-rank tests for male and female patients with primary breast lymphoma. (**A** and **B**) Before propensity scoring matching (PSM). (**C** and **D**) After PSM.

Next, we performed survival comparisons in 118 males and 2,496 females with PBL (4 males and 47 females died at diagnosis, respectively). As shown in Figure 2, significant differences in the survival outcomes were observed between male patients with PBL in terms of both OS and CSS. The 1-year, 5-year, and 10-year OS rates for males were 82.4%, 57.6%, and 37.3%, respectively, while the corresponding rates for females were 91.5%, 72.9%, and 54.5%, respectively (Figure 2A; *P* < 0.001). Furthermore, the 1-year, 5-year, and 10-year CSS rates of 91.1%, 72.2%, and 59.6% for males, respectively, were significantly lower than the corresponding rates of 94.4%, 86.4%, and 80.5%, for females, respectively (Figure 2B; *P* < 0.001).

In order to balance the clinical characteristics between the two groups of different gender, we further performed PSM analyses with a caliper value of 0.05 in a ratio of 1:2. The comparisons of demographics between them after matching are shown in Table 1 without any significant difference. The PSM results indicated that male patients with PBL indeed had significantly worse survival outcomes in terms of both OS (Figure 2C; *P* = 0.041) and CSS (Figure 2D; *P* = 0.002) than female patients.

### Prognostic Factors for Male Primary Breast Lymphoma

Univariate and multivariate Cox hazard proportional regression analyses were sequentially used for identifying the potential risk factors associated with OS among male patients with PBL (Table 2). The results revealed that patients' age [hazard ratio (HR) = 1.08, 95% CI: 1.05–1.12, *P* < 0.001] and radiotherapy (received vs. not received: HR = 0.41, 95% CI: 0.21–0.78, *P* = 0.007) significantly affected OS, which indicated that the risk of death increased by 8% (95% CI: 5%–12%) for each additional year of patients' age and decreased by 59% (95% CI: 22%–79%) when patients received radiotherapy.

**Table 2:** Univariate and multivariate Cox hazard proportional regression results in male patients with primary breast lymphoma.

Characteristic	Univariate Cox regression			Multivariate Cox regression		
	HR	95% CI	*P*-value	HR	95% CI	*P*-value
Age, years	1.07	1.04–1.10	**<0** **.** **001**	1.08	1.05–1.12	**<0**.**001**
Race						
White	Reference	—	—	Reference	—	—
Others	1.02	0.437–2.376	0.965	1.11	0.45–2.73	0.823
Year of diagnosis						
2000–2004	Reference	—	—	Reference	—	—
2005–2009	0.7	0.35–1.42	0.323	0.69	0.32–1.50	0.344
2010–2014	1.11	0.55–2.24	0.767	0.93	0.40–2.17	0.864
2015–2019	0.49	0.19–1.25	0.137	0.42	1.14–1.24	0.116
Subtype						
HL	Reference	—	—	Reference	—	—
NHL	2.03	0.28–14.7	0.483	1.62	0.19–13.45	1.616
Laterality						
Left	Reference	—	—	Reference	—	—
Right	0.91	0.58–1.42	0.677	1.09	0.61–1.94	0.657
Ann Arbor stage						
Stage I	Reference	—	—	Reference	—	—
Stage II	1.11	0.49–2.49	0.805	1.197	0.51–2.79	0.678
Stage III	1.97	0.67–5.80	0.22	0.67	0.21–2.19	0.508
Stage IV	2.42	1.19–4.94	**0**.**015**	0.938	0.37–2.40	0.893
Surgery						
Not received	Reference	—	—	Reference	—	—
Received	0.6	0.35–1.05	0.072	0.62	0.32–1.22	0.166
Radiotherapy						
Not received	Reference	—	—	Reference	—	—
Received	0.51	0.28–0.92	**0**.**026**	0.41	0.21–0.78	**0**.**007**
Chemotherapy						
Not received	Reference	—	—	Reference	—	—
Received	1.15	0.69–1.91	0.599	1.36	0.71–2.59	0.354

HR, hazard ratio; CI, confidence interval; HL, Hodgkin’s lymphoma; NHL, non-Hodgkin’s lymphoma.

Bold indicates *P* value < 0.05.

### Death Causes

**Figure 3:**
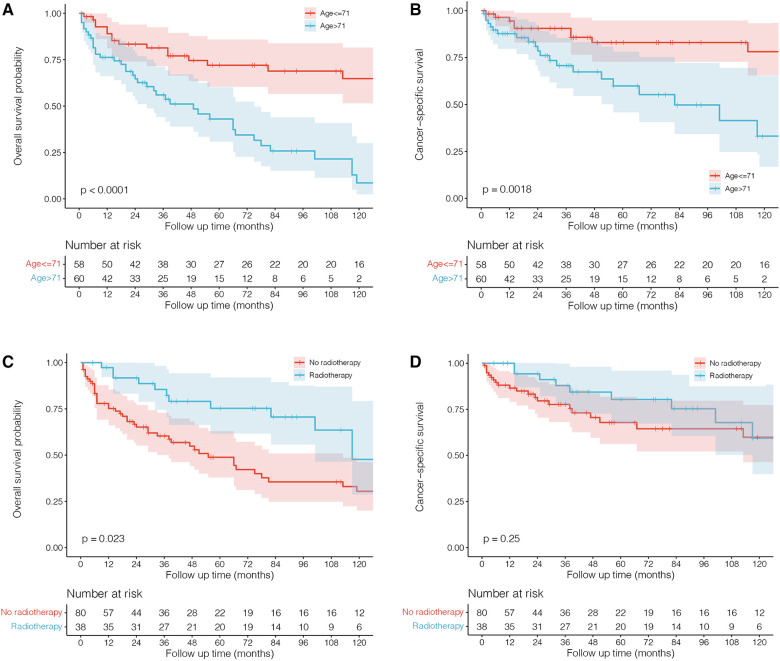
Death causes of male and female patients with primary breast lymphoma. HL, Hodgkin’s lymphoma; NHL, non-Hodgkin’s lymphoma.

Until the last follow-up update (November 2021), 1,005 (39.5%) females and 64 (52.5%) males had experienced death events among PBL patients from 2000 to 2019. Of female patients, 50.0% died of NHL, 4.7% died of HL, and 12.5% died of heart diseases. Of male patients, 38.6% died of NHL, 0.6% died of HL, and 13.8% died of heart diseases (Figure 3). The details of the causes of deaths in female and male patients are listed in **Supplementary Tables 1 and 2**, respectively.

## Discussion

Based on the initial definition by Wiseman and Liao(5), a diagnosis of PBL can be confirmed when lymphoma primarily occurs in the breast. As a rare disease, PBL constitutes a small proportion of approximately 0.5% among breast cancers (1) and also accounts for very limited percentages among lymphomas with less than 1% of NHL cases and 1.7%–3% of extranodal NHL, since HL primarily in the breast can be hardly observed (24). While females constitute a majority of PBL patients (95%–100%) (25), male patients are extremely few in number. Less than 50 cases of male patients have been reported, and none of the cohorts of male PBL has been analyzed in previous literature studies (11). Thus, this study first conducted a comprehensive clinical analysis of PBL with the largest cohort of 122 male patients taken from the SEER database who were diagnosed between 2000 and 2019. Not only did we investigate the clinical characteristics we also reported the incidence and survival outcome of the rare disease. Additionally, a total of 2,543 female patients diagnosed during the same period were analyzed for making comparisons with males.

The proportion of males among all PBL cases was 4.5% (122/2,665), further indicating the rarity of the disease in males. We first reported that the overall incidence rate of male PBL from 2000 to 2019 was 0.169 (95% CI: 0.140–2.203) per million persons, which is lower than that of females (1.59, 95% CI: 1.53–1.65). Incidence rates for males and females remained stable in the past 20 years (Figure 1). Incidence rates increased as patients' age increased and reached the highest level at the age range of 80–84 years for both sexes. In brief, although it represents a rare entity of breast carcinomas, PBL demonstrates obvious sex inclination and maintains a stable incidence in both female and male patients. The lower incidence rates of male PBL may partly lead to high possibilities of misdiagnosis and missed diagnosis of the disease.

Most clinical demographics showed similar distributions between female and male patients with PBL (Table 1). We also observed that male patients seemed to have a higher age than females [median: 71 (60–80) vs. 67 (57–77) years], though the difference was not significant (*P* = 0.055). Moreover, HL occurred rarely among both male and female patients, but remained significantly more common in males [male (2.5%, 3/122) vs. female (0.7%, 17/2,543), *P* = 0.025). The details of histological subclassifications of female and male patients are listed in **Supplementary Tables 3 and 4**, respectively. For both genders, consistent with the previous study (13), diffuse large B-cell lymphoma was the most common type, followed by extranodal marginal zone lymphoma (mucosa-associated lymphoid tissue type) and follicular lymphoma. Thus, we hypothesize that male PBL patients may be similar to females in terms of clinical demographics.

**Figure 4:**
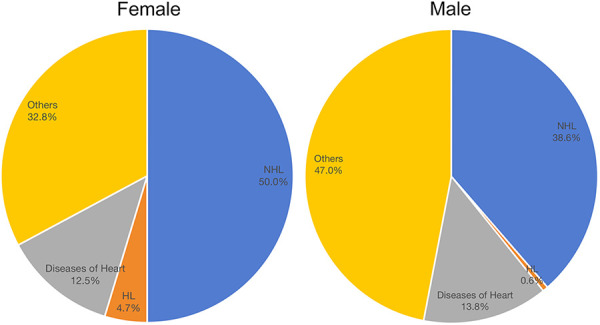
Kaplan–Meier survival curves and log-rank tests for male patients with primary breast lymphoma grouped by age and radiotherapy.

The standard treatment for male PBL remains unclear, and most of the reported cases were referred to the management for female patients (6–8, 10–20, 22, 23, 26). Our results demonstrated that chemotherapy and surgery did not significantly improve the survival outcome of male patients. However, radiotherapy significantly affected OS and decreased the death risk by 59% (95% CI: 22%–79%, *P* = 0.007), further confirming the efficacy of radiation on PBL, which could significantly improve local control of the disease (24). Jennings et al. (27) reviewed a large cohort of 465 patients with PBL and found that surgery such as mastectomy offered no survival benefits and did not improve recurrence. Lymph node status was also important in predicting survival rates and might be used to guide treatments such as chemotherapy and radiotherapy. Stage I patients with negative nodes showed good survival and recurrence rates when receiving radiation therapy, and stage II patients with positive nodes showed good survival and recurrence rates. However, there were very few male patients in their study, and a large cohort of male PBL is highly required to explore the potential therapeutics. Furthermore, survival analyses with the Kaplanâ€“Meier method also showed that male patients receiving radiotherapy had a better OS rate (*P* = 0.023) than those who did not receive this therapy, rather than CSS (which means males had a better OS but they did not have a better CSS), indicating its antitumor effects against PBL in males (Figure 4). In addition, we found that a higher age adversely affected survival outcomes, highlighting the importance of timely interventions for older males diagnosed with PBL.

To our knowledge, no cases of male cohorts with PBL have been reported before. Also, there is a lack of survival data. Our study first showed worse survival outcomes in males than in females with adjustments made for PSM (Figure 2). One of the possible causes for this is that male PBL is a rare occurrence and male patients are much more likely to suffer misdiagnosis or missed diagnosis, thus delaying appropriate treatments. In spite of the disease's low incidence rate, clinicians should exercise caution when a male patient presents with a breast lump. Biopsy may be necessary when it is difficult to make a definite diagnosis.

Although we reported the largest cohort of female PBL patients, there are several limitations in this study that need be highlighted. First, the patients in this study were included from the SEER database with retrospective data, and, therefore, bias may not be totally avoidable. Thus, we performed PSM analyses for making a more accurate comparison of survival outcomes between female and male patients. Second, there was a lack of detail on treatment regimens such as chemotherapy and radiotherapy in the SEER database. Third, systematic information on male PBL patients was also not recorded in the database, and therefore, this remained unanalyzed.

## Conclusion

Overall, we first reported a large cohort of male patients with PBL for making a comprehensive analysis. The incidence rate of 122 male patients diagnosed from 2000 to 2019 was 0.169 (95% CI: 0.140–2.203) per million persons, which was much lower than that of 2,543 females (1.59, 95% CI: 1.53–1.65). Clinical demographics were similar between females and males, except for lymphoma subtype distribution, and HL occurred more rarely in females, though it accounted for only a small proportion of all PBL patients. Meanwhile, we identified that higher age and not receiving radiotherapy were independent risk factors associated with OS in males. Radiotherapy can offer better survival benefits compared with surgery and chemotherapy. Using the PSM method, we also revealed that male PBL patients had significantly worse OS and CSS than females.

## Data Availability

The original contributions presented in the study are included in the article/**Supplementary Material**, further inquiries can be directed to the corresponding author.
