# Supplementary material for: Primary breast lymphoma in males: Incidence, demographics, prognostic factors, survival, and comparisons with females
Source: Front Surg. 2022 Aug 25;9:984497. doi: 10.3389/fsurg.2022.984497 (PMC9452836; doi:10.3389/fsurg.2022.984497)
Supplement: Supplementary file 1 [file Table_1_v2.docx]

**Supplement Table 1. Death causes of female patients with primary breast lymphoma.**

| **Death_cause_female** | **Number** | **Death_cause_female** | **Number** |
| --- | --- | --- | --- |
| Total | 1005 | Aleukemic, Subleukemic and NOS | 5 |
| Non-Hodgkin Lymphoma | 388 | Colon excluding Rectum | 5 |
| Diseases of Heart | 139 | Ovary | 5 |
| Other Cause of Death | 96 | Other Diseases of Arteries, Arterioles, Capillaries | 4 |
| Chronic Obstructive Pulmonary Disease and Allied Cond | 41 | In situ, benign or unknown behavior neoplasm | 3 |
| Breast | 36 | Liver | 3 |
| Cerebrovascular Diseases | 34 | Myeloma | 3 |
| Miscellaneous Malignant Cancer | 26 | Stomach | 3 |
| Alzheimers (ICD-9 and 10 only) | 24 | Symptoms, Signs and Ill-Defined Conditions | 3 |
| Lung and Bronchus | 23 | Uterus, NOS | 3 |
| Pneumonia and Influenza | 20 | Atherosclerosis | 2 |
| Diabetes Mellitus | 16 | Other Endocrine including Thymus | 2 |
| Septicemia | 13 | Other Lymphocytic Leukemia | 2 |
| State DC not available or state DC available but no COD | 13 | Soft Tissue including Heart | 2 |
| Other Infectious and Parasitic Diseases including HIV | 12 | Chronic Myeloid Leukemia | 1 |
| Chronic Lymphocytic Leukemia | 10 | Corpus Uteri | 1 |
| Accidents and Adverse Effects | 9 | Esophagus | 1 |
| Hypertension without Heart Disease | 8 | Gallbladder | 1 |
| Nephritis, Nephrotic Syndrome and Nephrosis | 7 | Intrahepatic Bile Duct | 1 |
| Brain and Other Nervous System | 6 | Melanoma of the Skin | 1 |
| Chronic Liver Disease and Cirrhosis | 6 | Non-Melanoma Skin | 1 |
| Hodgkin Lymphoma | 6 | Stomach and Duodenal Ulcers | 1 |
| Pancreas | 6 | Suicide and Self-Inflicted Injury | 1 |
| Acute Lymphocytic Leukemia | 5 | Thyroid | 1 |
| Acute Myeloid Leukemia | 5 | Urinary Bladder | 1 |
