# Supplementary material for: Primary breast lymphoma in males: Incidence, demographics, prognostic factors, survival, and comparisons with females
Source: Front Surg. 2022 Aug 25;9:984497. doi: 10.3389/fsurg.2022.984497 (PMC9452836; doi:10.3389/fsurg.2022.984497)
Supplement: Supplementary file 2 [file Table_2_v2.docx]

**Supplement Table 2. Death causes of male patients with primary breast lymphoma.**

| **Death_cause_male** | **Number** |
| --- | --- |
| Total | 64 |
| Non-Hodgkin Lymphoma | 32 |
| Diseases of Heart | 8 |
| Other Cause of Death | 5 |
| Hodgkin Lymphoma | 3 |
| Miscellaneous Malignant Cancer | 3 |
| Chronic Obstructive Pulmonary Disease and Allied Cond | 2 |
| Lung and Bronchus | 2 |
| Nephritis, Nephrotic Syndrome and Nephrosis | 2 |
| Chronic Liver Disease and Cirrhosis | 1 |
| Chronic Lymphocytic Leukemia | 1 |
| Congenital Anomalies | 1 |
| Esophagus | 1 |
| Other Infectious and Parasitic Diseases including HIV | 1 |
| Pneumonia and Influenza | 1 |
| Prostate | 1 |
