# Supplementary material for: Primary breast lymphoma in males: Incidence, demographics, prognostic factors, survival, and comparisons with females
Source: Front Surg. 2022 Aug 25;9:984497. doi: 10.3389/fsurg.2022.984497 (PMC9452836; doi:10.3389/fsurg.2022.984497)
Supplement: Supplementary file 3 [file Table_3_v2.docx]

**Supplement Table 3. Histological subclassifications of female patients with primary breast lymphoma.**

| **Lymphoid neoplasm recode 2021 for female PBL** | **Number** | **%** |
| --- | --- | --- |
| 1(a)1.2 Mixed cellularity | 4 | 0.16 |
| 1(a)2 Nodular sclerosis | 4 | 0.16 |
| 1(a)3 Classical Hodgkin lymphoma, NOS | 7 | 0.28 |
| 1(b) Nodular lymphocyte predominant Hodgkin lymphoma | 2 | 0.08 |
| 2(a)1 Precursor Non-Hodgkin lymphoma, B-cell | 3 | 0.12 |
| 2(a)2.1.1 Chronic/Small lymphocytic leuk/lymph | 97 | 3.81 |
| 2(a)2.1.3 Mantle-cell lymphoma | 31 | 1.22 |
| 2(a)2.2.1 Lymphoplasmacytic lymphoma | 18 | 0.71 |
| 2(a)2.3.1 DLBCL, NOS | 948 | 37.28 |
| 2(a)2.3.3 Primary effusion lymphoma | 1 | 0.04 |
| 2(a)2.4 Burkitt lymphoma/leukemia | 34 | 1.34 |
| 2(a)2.5.2 Extranodal MZL, MALT type | 628 | 24.70 |
| 2(a)2.6 Follicular lymphoma | 400 | 15.73 |
| 2(a)3 Non-Hodgkin lymphoma, B-cell, NOS | 183 | 7.20 |
| 2(b)1 Precursor Non-Hodgkin lymphoma, T-cell | 9 | 0.35 |
| 2(b)2.2.1 Peripheral T-cell lymphoma, NOS | 42 | 1.65 |
| 2(b)2.2.2 Angioimmunoblastic T-cell lymphoma | 4 | 0.16 |
| 2(b)2.2.3 Subcutan panniculitis-like T-cell lymph | 1 | 0.04 |
| 2(b)2.2.4 Anaplastic lar cell lymph, T-/Null-cell | 75 | 2.95 |
| 2(b)2.4 NK/T-cell lymph, nasal-type/aggres NK leuk | 3 | 0.12 |
| 2(c) Non-Hodgkin lymphoma, unknown lineage | 2 | 0.08 |
| 3 Composite Hodgkin lymphoma and NHL | 2 | 0.08 |
| 4 Lymphoid neoplasm, NOS | 45 | 1.77 |
