# Supplementary material for: Primary breast lymphoma in males: Incidence, demographics, prognostic factors, survival, and comparisons with females
Source: Front Surg. 2022 Aug 25;9:984497. doi: 10.3389/fsurg.2022.984497 (PMC9452836; doi:10.3389/fsurg.2022.984497)
Supplement: Supplementary file 4 [file Table_4_v2.docx]

**Supplement Table 4. Histological subclassifications of male patients with primary breast lymphoma.**

| **Lymphoid neoplasm recode 2021 for male PBL** | **Number** | **%** |
| --- | --- | --- |
| 1(a)2 Nodular sclerosis | 2 | 1.64 |
| 1(a)3 Classical Hodgkin lymphoma, NOS | 1 | 0.82 |
| 2(a)2.1.1 Chronic/Small lymphocytic leuk/lymph | 3 | 2.46 |
| 2(a)2.1.3 Mantle-cell lymphoma | 3 | 2.46 |
| 2(a)2.2.1 Lymphoplasmacytic lymphoma | 2 | 1.64 |
| 2(a)2.3.1 DLBCL, NOS | 45 | 36.89 |
| 2(a)2.5.2 Extranodal MZL, MALT type | 31 | 25.41 |
| 2(a)2.6 Follicular lymphoma | 21 | 17.21 |
| 2(a)3 Non-Hodgkin lymphoma, B-cell, NOS | 6 | 4.92 |
| 2(b)2.2.1 Peripheral T-cell lymphoma, NOS | 2 | 1.64 |
| 2(b)2.2.4 Anaplastic lar cell lymph, T-/Null-cell | 1 | 0.82 |
| 2(b)2.4 NK/T-cell lymph, nasal-type/aggres NK leuk | 1 | 0.82 |
| 4 Lymphoid neoplasm, NOS | 4 | 3.28 |
